# Relationship of vitamin D receptor expression with hormone receptors and other clinicopathological features in primary breast carcinomas: A retrospective cross-sectional study

**DOI:** 10.1097/MD.0000000000044222

**Published:** 2025-08-29

**Authors:** Yaşar Ünlü, Ethem Ömeroğlu, Abdülhalim Serden Ay, Meryem İlkay Eren Karanis, Kilinç Ayşe Nur Uğur, Esra Yilmaz

**Affiliations:** a University of Health Sciences Turkey, Konya City Hospital, Clinic of Pathology, Konya, Turkey; b University of Health Sciences Turkey, Konya City Hospital, Clinic of of General Surgery, Konya, Turkey.

**Keywords:** breast carcinoma, molecular subtyping, VDR

## Abstract

Breast cancer is a heterogeneous disease in which estrogen receptor (ER), progesterone receptor (PR), human epidermal growth factor receptor 2 (HER2), and Ki-67 play crucial roles in molecular subtyping, diagnosis, treatment, and prognosis, showing positivity in nearly 90% of cases. The vitamin D receptor (VDR) has been implicated in the oncogenesis and prognosis of various tumors, but its relationship with molecular subtyping factors in breast carcinomas remains to be clarified. This retrospective cross-sectional study included 111 patients who underwent surgery for breast carcinoma. Clinicopathological data were retrospectively analyzed in relation to VDR expression. Histological grade, hormone receptor (HR) status, Ki-67 proliferation index, and other clinicopathological parameters were recorded and their associations with VDR expression were statistically evaluated. Histological grading showed grade 1 in 3.7%, grade 2 in 41.4%, and grade 3 in 54.9% of cases. HR positivity was found in 60.3% of patients, while 39.7% were HR-negative. Ki-67 expression was ≥20% in 65.7% and <20% in 34.3% of cases. VDR expression was low in 54%, moderate in 26.1%, and high in 19.9% of patients. A strong correlation (*P* < .001) was observed between VDR expression and ER, PR, HR, and Ki-67, while a significant association (*P* = .025) was found with necrosis and mortality. Additionally, no significant correlation with histological grade (*P* = .056) was noted. The findings suggest a strong association between VDR and ER, PR status, and the Ki-67 proliferation index in breast carcinoma. Further studies are needed to explore the diagnostic, prognostic, and therapeutic implications of VDR in breast cancer.

## 1. Introduction

Breast cancer is the most common cancer among women worldwide, with approximately 2.3 million new diagnoses per year.^[1]^ It is the second most common cause of death after lung cancer.^[2]^ Breast cancer is a heterogeneous disease with various molecular characteristics.^[2,3]^ Many factors, including histological grade, tumor stage, and especially molecular subtype, are effective in prognosis.^[4]^ In molecular subtyping, 1 or more of the parameters estrogen receptor (ER), progesterone receptor (PR), human epidermal growth factor receptor 2 (HER2), and Ki-67 are expressed in approximately 90% of patients and play important roles in directing diagnosis and treatment.^[5]^ Molecular subtyping was developed by the St. Gallen International Expert Consensus in order to manage the treatment process better and with less budget,^[6]^ (Table 1). The clinical course and prognosis of triple-negative patients, which are seen in 10% to 20% of breast cancers, are quite poor.^[7]^ ER and HER2 positivity are seen in 70% to 75% and 15% to 20%, respectively, in breast cancers. Both antiestrogen and anti-HER2 therapeutic agents are used effectively. However, resistance develops in treatments applied in HER2-positive and up to 50% of ER-positive patients.^[8]^

**Table 1:** Molecular subtypes of breast cancer based on the 2013 St. Gallen International Expert Consensus definition.

	Luminal A	Luminal B HER2(−)	Luminal B HER2(+)	HER2 enriched	Triple negative
ER and PR	ER+ and/or PR+	ER+ and/or PR+	ER+ and/or PR+	ER− and PR−	ER− and PR−
HER2	HER2(−)	HER2(−)	HER2(+)	HER2(+)	HER2(−)
Ki-67%	Ki-67 ≤ 20%	Ki-67 > 20%	Any Ki-67	Any Ki-67	Any Ki-67

ER = estrogen receptor, HER2 = human epidermal growth factor receptor 2, PR = progesterone receptor.

Above 1.25(OH)2D3 is the active form of vitamin D and binds to the vitamin D receptor (VDR). With this binding, the receptor is transported to the nucleus and affects the regulation of more than 200 genes involved in cell growth, apoptosis, and cell signaling.^[2,9]^

VDR has been shown to be effective in the oncogenesis and prognosis of malignancies such as ovarian, thyroid, and colon cancer and melanoma.^[9]^ At the same time, there are many studies reporting that vitamin D inhibits cell proliferation, tumor invasion, metastasis, and angiogenesis and affects the induction of apoptosis and tumor cell differentiation.^[10]^ Various studies have demonstrated the effect of VDR on breast cell growth and immune regulation. Moreover, high VDR expression in breast carcinomas has been shown to be associated with better prognosis and survival.^[11]^ It has been stated that VDR blocks mitogenic activity driven by estrogen and also has an inverse relationship with breast carcinoma invasiveness.^[12,13]^ It has been said that activation of VDR is effective in both tamoxifen treatment and resistance development to this drug in ER-positive patients. Apart from VDR, it has been stated that vitamin D derivatives such as pure calcitriol reduce cancer risk and make significant contributions to treatments.^[14]^ In this study, the purpose was to investigate the relationship between VDR expression in breast carcinomas and molecular subtyping and other clinicopathological parameters that are widely used in the diagnosis and treatment of breast carcinoma. We hypothesized that low VDR expression correlates with worse clinicopathological features.

## 2. Materials and methods

### 2.1. Study population and subject

**Figure 1.**
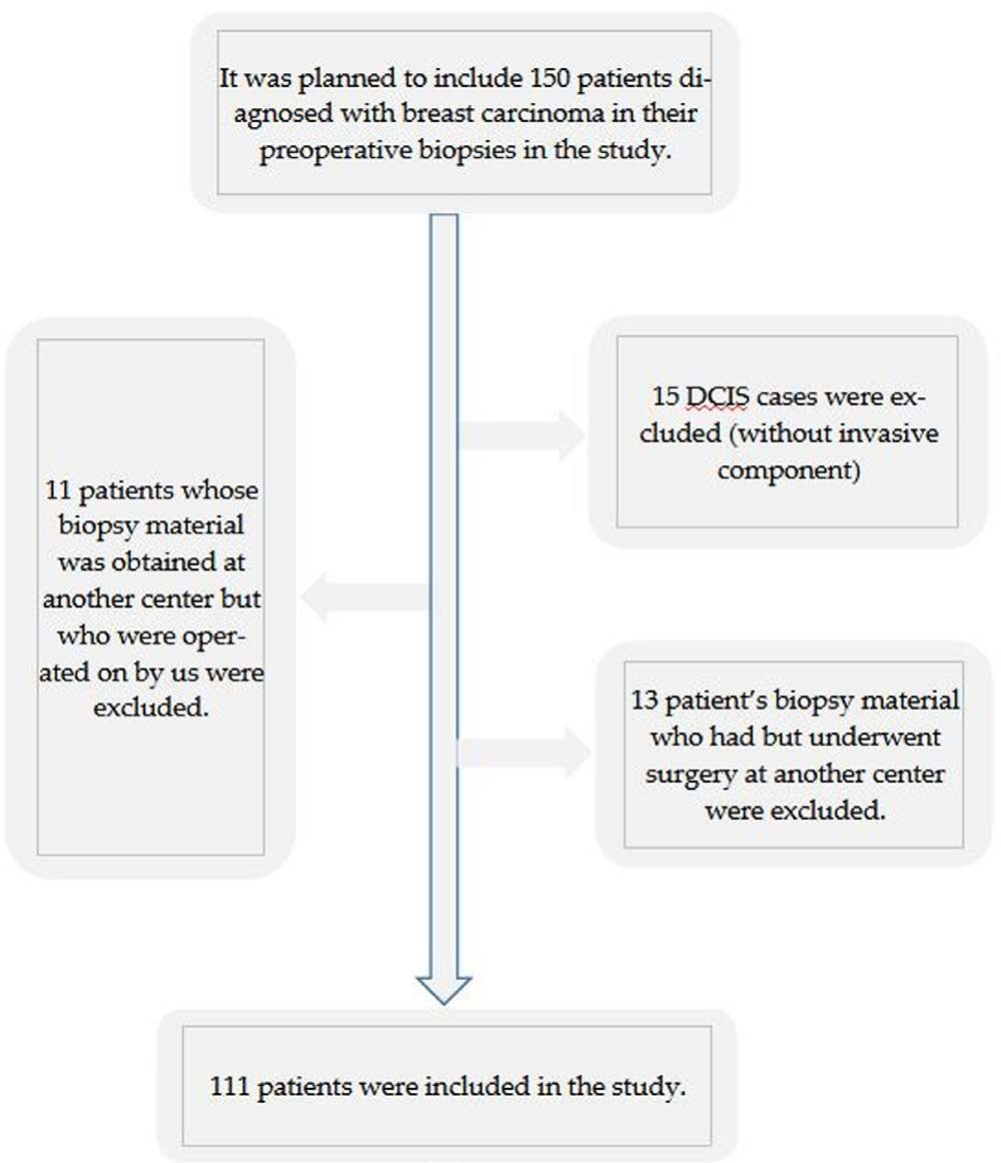
Flow chart of the study. DCIS = ductal carcinoma in situ.

This retrospective cross-sectional study was conducted at Konya City Hospital. Patients who were diagnosed with breast cancer and operated on between September 2020 and March 2023 at Konya City Hospital were included in this study. In the initial stage, 150 patients diagnosed with breast cancer and not considered for neoadjuvant systemic therapy (NST) were identified and the study was planned. Patients who lacked preoperative biopsy and postoperative surgical specimens in our hospital, whose specimens did not contain invasive breast carcinoma areas, or who had received NST were excluded from the study. Cases with available preoperative and postoperative pathological specimens in our institution containing invasive tumor areas, and who had not received NST, were included in the study. However, of these 150 patients, 15 patients who were diagnosed with only ductal carcinoma in situ without invasive components were not included in the study. Additionally, 13 patients who underwent surgery at another center after being diagnosed with breast cancer were excluded from the study. Furthermore, although surgery was performed at our center, 11 patients diagnosed at an external institution were not included due to a lack of access to their data from the referring center. No sampling method was used in the study. A total of 111 individuals who met the inclusion criteria during the study period constituted the sample size (Fig. 1). Following data collection, a retrospective power analysis was conducted using the GPower 3.1.9.4 software. In this analysis, Cohen’s standard effect size was accepted as 0.25 (medium; Cohen, 1992). The calculated minimum required sample size was 103 patients (*d* = 0.25, α = 0.05, 1 − β = 0.80). The final study population of 111 patients exceeded this required sample size, ensuring sufficient power for the analyses. Demographic data such as age, gender, and survival, and clinical data such as distant metastasis and other malignancies, if any, were obtained from the hospital registry system. This retrospective study was approved by the Local Ethics Committee of KTO Karatay University Faculty of Medicine (IRB: 2025/016, approval date: 30.01.2025). As the study was based on retrospective evaluation of previously collected clinical and pathological data, it was not registered in a clinical trial registry in accordance with institutional and national guidelines for non-interventional retrospective studies.

### 2.2. Evaluation of pathological findings and molecular subtyping parameters

**Figure 2.**
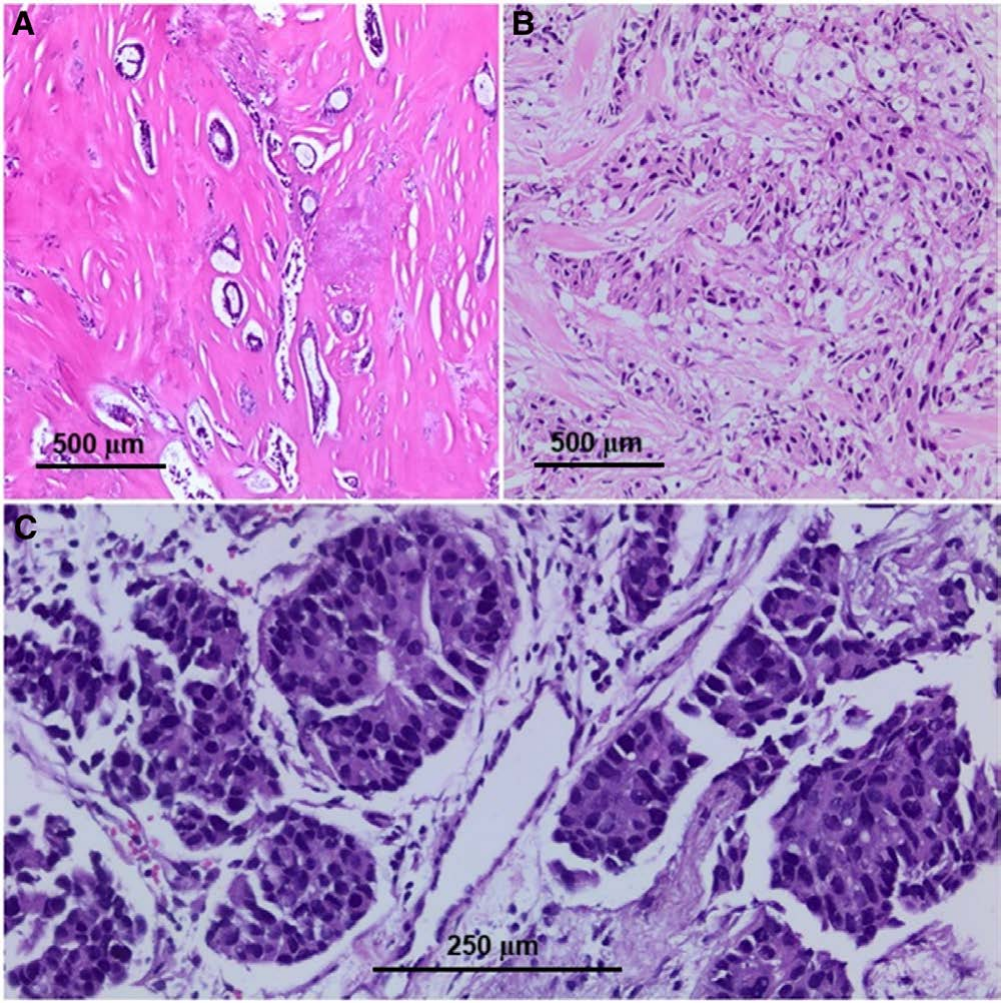
Histological grade of breast carcinoma: (A) grade 1 HEX100; (B) grade 2, HE × 100; and (C) grade 3, HE × 200.

**Figure 3.**
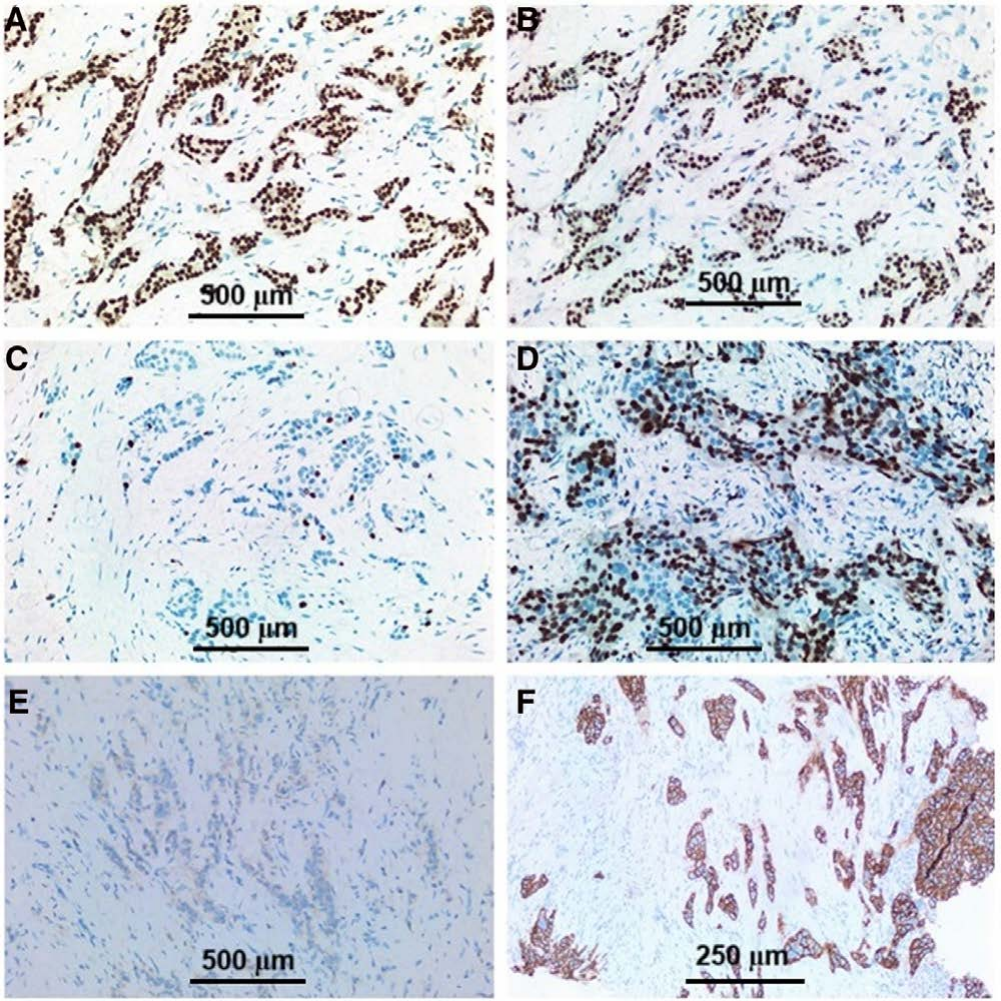
Immunohistochemical detection of ER, PR, Ki-67 and HER2 expression of breast carcinoma: (A) ER positive, ER × 100; (B) PR positive, PR × 100, (C) Ki-67; <20%, Ki-67 × 100; (D) Ki-67; >20%, Ki-67 × 100; (E) HER2 score 0, HER2 × 100; and (F) HER2 score 3, HER2 × 200. ER = estrogen receptor, PR = progesterone receptor, HER2 = human epidermal growth factor receptor 2.

Hematoxylin and eosin staining and immunohistochemical staining preparations containing ER, PR, Ki-67, and HER2 obtained from breast specimens and biopsies of the cases were reevaluated. In this context, histological subtype, histological grading (Fig. 2), tumor stage, tumor diameter, presence of necrosis and in situ carcinoma, and invasion of lymphovascular, perineural, surgical margins, and lymph nodes were evaluated. The Ki-67 proliferation rate was categorized as <20% (low) and ≥20% (high; Fig. 3).^[15]^ HER2 score evaluation was carried out using the ASCO/CAP guidelines for breast carcinoma. Zero and +1 were considered negative, and +3 were considered positive. Those with +2 were evaluated as positive or negative according to the presence or absence of amplification as a result of the FISH study.^[16]^ According to the 12th St. Gallen International Breast Cancer Conference (2011), breast cancer was molecularly divided into 5 subtypes: luminal A, luminal B, luminal B HER2 type, HER2 overexpression type, and triple-negative type,^[6]^ (Table 1; Fig. 3).

### 2.3. VDR immunohistochemistry staining and scoring

**Figure 4.**
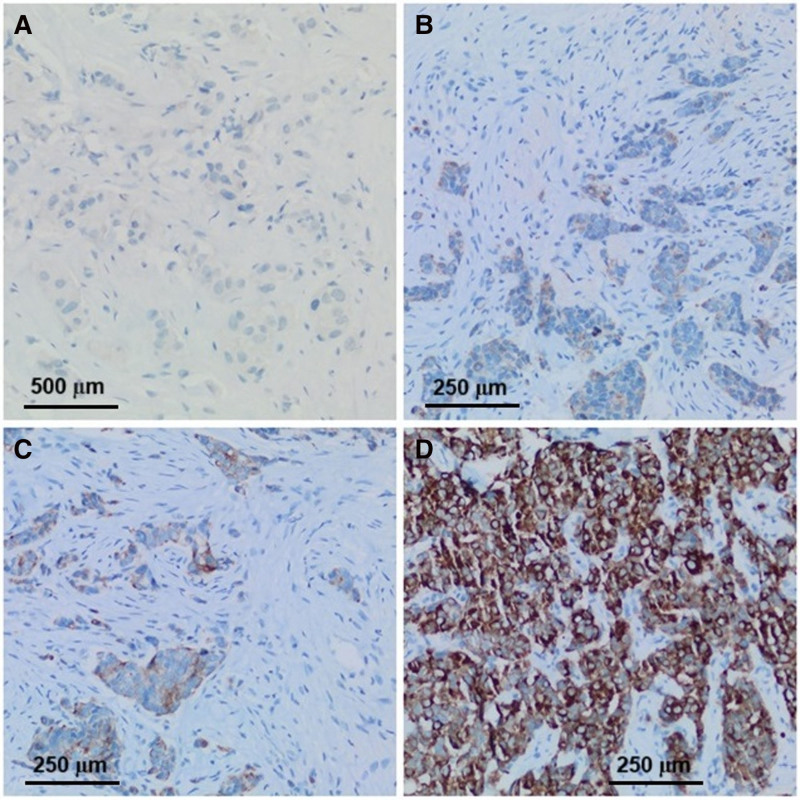
Immunohistochemical detection of VDR expression in breast carcinoma: (A) score 0, VDR × 100; (B) score 4, VDR × 200; (C) score 6, VDR, VDR × 200; and (D) score 9, VDR × 200. VDR = vitamin D receptor.

Paraffin blocks of preoperative biopsies of the patients were cut at 4-micron thickness and immunohistochemically stained with mouse anti-VDR/VDR pol antibody (1:100 diluted, Medaysis, Cat No. MCD304, Livermore). Primary antibodies were visualized by using the avidin-biotin-peroxidase complex method. Diaminobenzidine was used as the substrate. Sections were counterstained by Mayer’s hematoxylin. In slides that were stained with VDR, nucleus, cytoplasmic and membranous staining were considered positive. Staining percentage scoring (no staining: 0–25%:1, 26–50%:2, >50%:3) and staining intensity scoring (no staining: 0, mild: 1, moderate: 2, and strong: 3) were determined.^[17]^ The results of the multiplication of the above 2 parameters were performed as Total Scoring (negative; 0, mild; <4, moderate; 4–6, and strong; +7) similar to previous studies (Fig. 4).^[18]^

### 2.4. Statistical analysis

Statistical analyses were conducted by using the IBM SPSS software version 20.0 (SPSS Inc., Chicago). Categorical variables were evaluated using the chi-square test. The normality of the distribution was assessed using the Kolmogorov–Smirnov test. For comparisons involving 3 or more groups, 1-way ANOVA was performed in cases of parametric distribution, and when a significant difference was detected, post hoc analyses were conducted using Tukey’s test. A *P*-value of <.05 was considered statistically significant. As this was a retrospective study, selection bias cannot be entirely ruled out; however, all cases were included consecutively.

## 3. Results

### 3.1. Clinicopathological features

The study included 111 female patients diagnosed with breast carcinoma. The mean age was 56.37 ± 13.04. Of the cases, 103 (92.7%) had invasive ductal carcinoma and 8 (7.3%) had invasive lobular carcinoma. The histological grades of the patients were as follows: grade 1 was 4 (3.7%), grade 2 was 46 (41.4%), and grade 3 was 61 (54.9%). Hormone receptor positive was 67 (60.3%) and negative was 44 (39.7%). Ki-67 ≥ 20% was 73 (65.7%), and <20% was 38 (34.3%). The distribution of the patients based on molecular subtyping is as follows: luminal A 16 (14.5%), luminal B HER2(−) 21 (18.9%), luminal B HER2(+) 30 (27%), HER2 overexpression 20 (18%), and triple negative 24 (21.6%). Immunohistochemically applied VDR results showed low expression in 60 (54%), medium expression in 29 (26.1%) and high expression in 22 (19.9%) of the cases. These mentioned and not mentioned relevant clinicopathological findings are summarized in Table 2.

**Table 2:** Characteristics of the patients.

N = 111*						
Gender		Female	111 (100%)	Stage T	T1	28 (25.2%)
		Male	0 (0%)		T2	68 (61.2%)
Mortality		Present	14 (12.7%)		T3	15 (13.6%)
		Absent	97 (87.3%)	ER	Positive	64 (57.6%)
Laterality		Right	51 (46%)		Negative	47 (42.4%)
		Left	60 (54%)	PR	Positive	48 (43.3%)
Focality		1	89 (80.1%)		Negative	63 (56.7%)
		≥2	22 (19.9%)	AR	Positive	21 (67.7%)
Histological type		IDC	103 (92.7%)		Negative	10 (32.3%)
		ILC	8 (7.3%)	HR status	Positive	67 (60.3%)
Histological grade		1	4 (3.7%)		Negative	44 (39.7%)
		2	46 (41.4%)	Ki-67 index	≥20%	73 (65.7%)
		3	61(54.9%)		<20%	38 (34.3%)
LVI		Present	47 (42.4%)	HER2 status	Positive	49 (44.2%)
		Absent	64 (57.6%)		Negative	62 (55.8%)
PNI		Present	20 (18.1%)	Molecular subtype	Luminal A	16 (14.5%)
		Absent	91 (81.9%)		Luminal B HER2(−)	21 (18.9%)
SMI		Present	3 (2.8%)		Luminal B HER2(+)	30 (27%)
		Absent	108 (97.2%)		HER over exspresion	20 (18%)
Necrosis		Present	19 (17.2 %)		Triple-negative	24 (21.6%)
		Absent	92 (82.8%)	VDR score	High	22 (19.9%)
Carcinoma in situ	Present		44 (39.7%)		Mediim	29 (26.1%)
	Absent		67 (60.3%)		Low	60 (54%)
Lymph node metastasis	Present		53 (47.8%)			
	Absent		58 (52.2%)			

AR = androgen receptor, ER = estrogen receptor, HER2 = human epidermal growth factor receptor 2, HR = hormone receptor, IDC = invasive ductal carcinoma, ILC = invasive lobular carcinoma, LVI = lymphovascular invasion, PNI = perineural invasion, PR = progesterone receptor, SMI = surgical margin involvement, T = tumor, VDR = vitamin D receptor.

* n (%); mean ± SD; median (25–75%).

### 3.2. VDR expression patterns

A significant relationship was found in those with low VDR expression, with higher mortality (*P* = .025). Higher histological grade and necrosis were detected in those with low VDR expression. A relationship was observed with a *P*-value of .056, which did not reach statistical significance. No significant relationship was found between other clinicopathological findings and VDR expression (*P* > .05; Table 3). Striking results were obtained between VDR expression and molecular subtyping and parameters.

**Table 3:** VDR expression and clinicopathological features in patients with breast carcinoma.

Feature	Category	VDR score			*P*-value
		Low	Medium	High	
Laterality	Right	31	12	8	.397
	Left	29	17	14	
Mortality	Present	12	0	2	.025
	Absent	48	29	20	
Focality	1	51	21	17	.372
	≥2	9	8	5	
Histological grade	1	2	2	0	.056
	2	18	15	13	
	3	40	12	9	
LVI	Present	27	11	9	.809
	Absent	33	18	13	
PNI	Present	10	5	5	.826
	Absent	50	24	17	
SMI	Present	0	1	2	.076
	Absent	60	28	20	
Necrosis	Present	15	2	2	.056
	Absent	45	27	20	
Carcinoma in situ	Present	22	13	9	.755
	Absent	38	16	13	
Stage T	T1	10	11	7	.062
	T2	39	14	15	
	T3	11	4	0	
Lymph node metastasis	Present	34	10	9	.127
	Absent	26	19	13	

LVI = lymphovascular invasion, PNI = perineural invasion, SMI = surgical margin involvement, T = tumor, VDR = vitamin D receptor.

### 3.3. Correlation with moleculer subtype parameters

According to ER, PR, and hormone receptor status, a significant relationship was found in those with negative VDR expression scores, mostly low VDR expression, while positive VDR expression scores were found to be moderate and high (*P* < .001). No significant relationship was found between AR and HER2 status (*P* > .05). While those with triple-negative and HER2 overexpression molecular subtyping parameters showed low VDR expression, other groups such as luminal A and luminal B showed moderate and high VDR expression. A significant relationship was found between luminal B HER2(+) 30 (27%) and VDR expression (*P* < .001). While most of those with Ki-67 index ≥20% showed low VDR expression, those with Ki-67 index <20% showed moderate and high VDR expression. Thus, a highly significant relationship (*P* < .001) was detected between the Ki-67 index and VDR expression (Table 4).

**Table 4:** VDR expression and HR, HER2, and molecular subtype in patients with breast carcinoma.

Feature	Category	VDR score			*P*-value
		Low	Medium	High	
ER	Positive	18	24	22	<.001
	Negative	42	5	0	
PR	Positive	14	16	18	<.001
	Negative	46	13	4	
AR	Positive	9	8	4	.644
	Negative	6	3	1	
HR status	Positive	21	24	22	<.001
	Negative	39	5	0	
Ki-67 index	≥20%	48	17	8	<.001
	<20%	12	12	14	
Her2 status	Positive	24	16	9	.880
	Negative	36	13	13	
Molecular subtype	Luminal A	5	6	5	<.001
	Luminal B HER2(−)	7	6	8	
	Luminal B HER2(+)	9	12	9	
	HER over exspresion	16	4	0	
	Triple-negative	23	1	0	

AR = androgen receptor, ER = estrogen receptor, HER2 = human epidermal growth factor receptor 2, HR = hormone receptor, PR = progesterone receptor, VDR = vitamin D receptor.

## 4. Discussion

There are numerous studies investigating the relationship between breast carcinoma and VDR. These studies have generally been conducted using resection specimens, animal models, and molecular applications. In our study, only preoperative biopsy specimens were used. In this respect, it is one of the pioneering publications aiming to investigate the relationship between VDR expression in tumor cells in preoperative breast biopsy specimens and prognostic indicators.

Breast cancer is the most common type of cancer among women worldwide. While early-stage and nonmetastatic cases can be treated at a rate of 70% to 80%, the prognosis is worse in advanced-stage and metastatic cases.^[10]^ Although there are some publications reporting different results, most of the studies on VDR in breast carcinoma regarding prognosis and treatment processes have demonstrated that tumors with low VDR expression are associated with a poorer prognosis.^[19]^ In a study by Li et al including 581 patients, it was shown that patients with high VDR expression in ER-positive breast cancer had better survival outcomes.^[20]^ Ditsch et al conducted a study with 82 primary breast carcinoma patients and found a strong correlation between VDR expression and overall survival and disease-free survival (*P* = .014 and *P* < .037, respectively).^[21]^ In contrast to these results, a study by Al-Azhri et al found no significant association between VDR expression and survival.^[22]^ In our study, a significant relationship was found, indicating higher mortality in cases with low VDR expression (*P* = .026). Larger case series and 5- or 10-year survival studies would be more beneficial to clarify these results.

In our study, while a significant relationship was observed between VDR and prognostic factors of breast carcinoma such as ER, PR, and Ki-67 (*P* < .001), no significant relationship was found with HER2 (*P* > .05).

### 4.1. VDR is a member of the human nuclear receptor superfamily, like ER and PR^[5]^

In addition to the known role of ERs in the pathogenesis, diagnosis, and treatment of breast cancer, close interactions between VDR and ERs have also been identified.^[23]^ In 1 study, estrogen-related receptor alpha was shown to play an important role in breast carcinoma oncogenesis, with VDR and estrogen-related receptor alpha acting jointly at the intersection of estrogen signaling activation.^[14]^ In a study conducted by Dogra et al, a significant relationship was found between VDR expression and ER, PR, and HER2 (*P* < .001).^[23]^ In a study conducted by Dai et al with 2433 patients, a positive correlation was detected between VDR and both ER and HER2, while no relationship was found with PR.^[24]^ Hemida et al demonstrated a correlation between tissue VDR and ER-α expressions.^[25]^ In an experimental study, a significant relationship was found between VDR and both ER and HER2, while no VDR expression was observed in Ki-67 positive cells.^[26]^

In contrast to these studies, no correlation was found between VDR and ER, PR, and HER2 in 2 separate studies conducted by Didiagnos et al^[27]^ and Bahador et al.^[28]^ In a study by Kimondo et al with 214 patients, no significant relationship was observed between VDR expression and ER or PR, while a weak correlation was found with HER2.^[29]^

In our study, mostly low VDR expression was found in patients negative for ER, PR, and HER2, whereas intermediate and high VDR scores were observed in positive patients, and a statistically significant relationship was detected between VDR and molecular subtypes (*P* < .001). Studies involving molecular subtyping in breast carcinomas have generally yielded similar results. In the previously mentioned study by Al-Azhri et al, a significant relationship was found between VDR expression and ER, PR, Ki-67, and molecular subtypes in 1114 patients, but no association was found between HER2 and VDR expression.^[22]^ In another study conducted by Putri et al with 75 patients, a significant relationship was reported between luminal and non-luminal groups in terms of VDR expression (*P* = .047).^[30]^ In a study by Horas et al involving 171 patients, high VDR expression was observed in luminal A and B subtypes, while low expression was seen in triple-negative patients.^[31]^ The findings of the above studies generally support our results. In contrast, in a meta-analysis study by Haiyan et al involving 1585 patients, no significant relationship was found between molecular subtyping and VDR expression.^[32]^

As can be understood from the aforementioned study results, different findings can be encountered in studies investigating the relationship between various prognostic parameters and VDR expression in breast cancer cases. The most important reasons for this are thought to be VDR polymorphism, tumor heterogeneity, and downregulation or ablation of VDR during tumor progression.^[2,22]^

In the literature, apart from the relationship between VDR expression and molecular subtypes, studies investigating the association of VDR with other pathological parameters also exist. The results regarding the relationships between VDR and these pathological parameters have varied. In our study, no statistically significant relationship was found between VDR expression and tumor necrosis or histological grade (*P* = .056), as well as with other pathological findings such as T stage and lymphovascular invasion (*P* > .05).

In a study by Huss et al involving 878 breast carcinoma patients, a significant relationship was found between high VDR expression and lower tumor grades. In the same study, it was reported that VDR-negative patients required neoadjuvant therapy more frequently.^[33]^ In 2 previously mentioned studies by Hemida and Horas, higher VDR expression was observed in lower-grade breast carcinomas.^[25,31]^ In a study by Nam et al involving 1012 breast cancer patients, a significant association was detected between VDR expression and TNM staging (*P* < .05).^[34]^ A study by Wanda et al with 49 patients demonstrated a negative correlation between tumor stage and VDR expression.^[4]^ Similarly, 3 separate studies by Zilenaite, Kimondo, and Putri reported a significant relationship between tumor stage and VDR.^[15,29,30]^

In contrast to the above study results, no significant relationship was found between VDR values and tumor stage or histological grade in a study conducted by Dai et al involving 2433 patients.^[24]^ In another study involving 50 patients, no relationship was detected between VDR and tumor stage, lymphovascular invasion, or lymph node involvement.^[28]^ The results of these last 2 studies are in line with our findings.

It has been shown that VDR expression in tumor tissues affects tamoxifen resistance in breast carcinomas,^[20]^ and that VDR agonists such as calcitriol positively contribute to the treatment of both ER-negative^[35]^ and ER-positive^[20]^ breast cancers, increasing the antiproliferative effect of 5-fluorouracil even at low doses.^[36]^ Moreover, it has been reported to have a preventive role in the development of breast cancer.^[37]^ Additionally, beneficial effects of vitamin D supplementation have been mentioned in patients scheduled for NST.^[38]^ In future prospective studies, comparing VDR expression results in preoperative biopsy specimens and residual tumors in resection specimens after neoadjuvant therapy could provide more valuable results regarding the therapeutic effect of VDR in breast cancers. The significant findings obtained in our study between VDR expression and triple-negative patients as well as ER, PR, and Ki-67 results may offer beneficial approaches for the management of breast carcinomas at all stages.

Serum vitamin D levels and VDR expressions in cancerous tissues do not always show parallelism due to mutations and polymorphisms in the receptor.^[11,27]^ This study has several limitations. First, due to its retrospective cross-sectional design, causality cannot be established. Second, although consecutive cases were included to reduce selection bias, some degree of sample bias may still exist. Third, the single-center nature of the study limits the generalizability of the findings to broader populations. Fourth, the evaluation of VDR expression was based solely on immunohistochemistry, which may be subject to interobserver variability and technical limitations.

## 5. Conclusion

In our study, VDR expression was evaluated in preoperative biopsy specimens of patients who underwent surgery for breast carcinoma. A significant relationship was found between VDR and ER, PR status, and the Ki-67 proliferation index in breast carcinoma cases. Additionally, the association between VDR and molecular subtypes was demonstrated. In the future, larger series studies including serum vitamin D levels and VDR expression findings in residual tumor tissues after NST are recommended. We believe that the results obtained by combining the findings of these studies with molecular and therapeutic parameters will contribute more effectively to the prognosis and treatment of breast cancer.

## Acknowledgments

The authors would like to express their sincere gratitude to Mehmet Sinan İyisoy from the Department of Biostatistics, Necmettin Erbakan University, for his valuable contributions and assistance with the statistical analyses of this study.

## Author contributions

**Conceptualization:** Yaşar Ünlü.

**Data curation:** Ethem Ömeroğlu.

**Formal analysis:** Ethem Ömeroğlu.

**Investigation:** Abdülhalim Serden Ay.

**Methodology:** Abdülhalim Serden Ay.

**Project administration:** Mİ Eren Karanis.

**Software:** Aysenur U. Kilinc.

**Supervision:** Aysenur U. Kilinc.

**Validation:** Esra Yilmaz.

**Visualization:** Esra Yilmaz.

**Writing** – **original draft:** Yaşar Ünlü.

**Writing** – **review & editing:** Yaşar Ünlü.
